# How Do Poverty, Insurance, and Government Assistance Impact Diabetic Foot Ulcer Outcomes? A Scoping Review

**DOI:** 10.1002/dmrr.70219

**Published:** 2026-08-21

**Authors:** Jamie N. LaMantia, Devan McClain, Abigail Daniele, Michael S. Conte, Maya Fayfman, Alisha Oropallo, Marcos C. Schechter, Cathie Spino, Wen Ye, James C. Iannuzzi, Meghan B. Brennan

**Affiliations:** ^1^ Division of Infectious Disease, Department of Medicine, School of Medicine and Public Health University of Wisconsin Madison Wisconsin USA; ^2^ School of Integrative Biology University of Illinois Urbana Illinois USA; ^3^ Division of Vascular and Endovascular Surgery, Department of Surgery University of California San Francisco San Francisco California USA; ^4^ Division of Endocrinology, Metabolism and Lipids, Department of Medicine Emory University School of Medicine Atlanta Georgia USA; ^5^ Grady Memorial Hospital Atlanta Georgia USA; ^6^ Department of Vascular Surgery Donald and Barbara Zucker School of Medicine, Hofstra/Northwell Hempstead New York USA; ^7^ Division of Infectious Disease, Department of Medicine Emory University School of Medicine Atlanta Georgia USA; ^8^ Department of Biostatistics University of Michigan School of Public Health Ann Arbor Michigan USA

**Keywords:** amputation, causal pathway, diabetic foot ulcer, limb salvage, mediation, moderation, social drivers of health

## Abstract

**Aims:**

The social drivers of diabetic foot disease (SDDFD) impact ulcer incidence and outcomes, but *how* is relatively unknown. We aimed to build a conceptual model positing how three SDDFD—poverty, insurance, and government assistance—influence outcomes for patients with diabetic foot ulcers.

**Materials and Methods:**

To inform the model, we conducted a scoping review of studies linking poverty, insurance, and/or government assistance with diabetic foot ulcer outcomes using Levac and colleagues' 6‐stage framework, the Preferred Reporting Items for Systematic Review and Meta‐analysis extension for scoping reviews, and the SPIDER tool. We searched PubMed, CINAHL, and Embase for original research articles set in the United States and published between 1 January 2005 and 7 November 2025. We excluded those published in a language other than English, case reports, abstracts, and studies of other SDDFD.

**Results:**

Data from 35 included studies informed our conceptual model, depicting a complex causal pathway between SDDFD and outcomes, often mediated by healthcare system factors and moderated by patients' abilities to adhere to healthcare recommendations. Multidisciplinary teams might ameliorate the risk of amputations associated with poverty, lack of insurance, or qualifying for government assistance. Additionally, the model highlights reinforcing feedback loops, both positive and negative.

**Conclusion:**

Our model can be used to inform mediation and moderation analyses and help avoid collider‐conditioning bias during statistical analyses. It can help identify potential points of intervention within the healthcare system for those working to dampen the negative effects of SDDFD on outcomes.

## 1 Introduction

All models are wrong, but some are useful.

∼ George Edward Pelham Box, statistician

The burden of diabetic foot ulcers, experienced by over 1.5 million people in the United States each year, is inequitable [1]. Disparities exist along nearly every investigated social line [2]. Patients identifying as a racial or ethnic minority, living in rural areas, or residing in disadvantaged neighborhoods consistently experience worse outcomes, including greater major amputation risks (i.e., above‐ or below‐knee amputations) [3, 4, 5, 6, 7]. We have known this for decades. What we have yet to fully understand is *how* social drivers impact ulcer outcomes.

A clearer understanding of the causal pathway between social drivers of diabetic foot disease (SDDFD) and ulcer outcomes is likely to support better design and evaluation of interventions aimed at mitigating disparities. A conceptual model would help identify interactions between variables along the causal pathway, which would allow statistical models to account for mediators, moderators, and colliders. Those models would then more precisely estimate the effect of intervening at a variable in the pathway. Here, we aim to build a conceptual model positing how SDDFD—specifically, poverty, insurance, and government assistance—impact diabetic foot ulcer outcomes in the contemporary United States. We focus specifically on poverty, insurance, and government assistance because we think these are more proximal to the outcome than other SDDFD, such as race; proximal drivers are likely to have clearer associations with the outcome, allowing researchers to work from proximal to distal drivers and expand the scope of the conceptual model.

We conducted a scoping review to inform our conceptual model. Scoping reviews facilitate a ‘mapping’ process to convey the breadth of evidence on a specific health topic [8]. They promote analytic reinterpretation of the literature and often culminate in the synthesis of findings from different study types [9]. A scoping review was an appropriate approach because we wanted to: (1) capture a broad understanding of how three proximal SDDFD influence foot ulcer outcomes; (2) include both quantitative and qualitative studies; and (3) build a conceptual model that could inform future statistical analyses and identify points of intervention to address amputation disparities.

In attempting this work, we recognise that it may be impossible to completely parse out the complex interplay between SDDFD and ulcer outcomes [10, 11, 12]. Models are incomplete simplifications of complex phenomena. Our model will be wrong. However, we hope that it will be useful for building statistical regressions, interpreting results, and driving evidence‐based hypotheses about what will happen when we intervene on variables in the model.

## 2 Materials and Methods

We used four sources to improve the rigour of our work. First, the 6‐step framework proposed by Levac and colleagues provided an overarching approach [8]. We omitted the optional sixth step (consulting with stakeholders) because our team benefits from representation across multiple groups who would be end‐users of this product. Specifically, co‐authors include practicing physicians specialising in diabetic foot ulcers from endocrinology, infectious disease, and vascular surgery. Second, we used the Sample, Phenomenon of Interest, Design, Evaluation, Research type (SPIDER) tool to hone our research question [13]. Third, we referenced the Joanna Briggs Institute (JBI) Manual for Evidence Synthesis when developing our search strategy, building our data extraction tool, and synthesising data to build our conceptual model [14]. Finally, we followed the Preferred Reporting Items for Systematic Review and Meta‐analysis extension for scoping reviews (PRISMA‐ScR) to present our findings [15]. Requests for the full review protocol should be sent to the corresponding author.

### 2.1 Framework Stage 1: Identifying the Review Question

We developed the review question based on our target population (patients with diabetic foot ulcers), the outcome of interest (ulcer outcomes), and the concept of interest (*how* poverty, insurance, and government assistance impact outcomes) [8]. We hypothesised that poverty, insurance, and government assistance were some of the most proximal SDDFD and limited our scoping review to these drivers, presuming that other social drivers such as race and gender would be more distal to ulcer outcomes. We used the SPIDER tool to hone our questions because we wanted to include quantitative, qualitative, and mixed methods studies [13]. This led to our final question: How do poverty, health insurance, and government assistance influence limb salvage for contemporary patients with diabetic foot ulcers living in the United States? We limited our scope to the United States because of its unique social and insurance pressures operating outside a universal healthcare system.

### 2.2 Framework Stage 2: Identifying Relevant Studies

We searched PubMed, Cumulative Index to Nursing and Allied Health Literature (CINAHL), and Embase using terms refined with the help of a reference librarian (Supporting Information S1: Appendix 1). Articles indexed 1 January 2005 through 7 November 2025 (last day of search) were included to maximise contemporary relevance and capture the U.S. insurance landscape following the Medicare Modernisation and Affordable Care Acts. Additional inclusion criteria were as follows: original research article, conducted in the United States, included information on at least one of our three social drivers (poverty, health insurance, and government assistance), and its impact on diabetic foot ulcer outcomes, including major amputations, minor amputations, healing, worsening ulcer severity, and the need for hospitalisation. Exclusion criteria included case reports and reviews, abstract‐only material, animal studies, not specific to patients with diabetic foot ulcers, and studies written in a language other than English. Finally, we manually searched the references of included studies culled from database searches to find additional studies meeting our inclusion/exclusion criteria.

### 2.3 Framework Stage 3: Study Selection

After duplicates were removed, J.N.L. initially screened articles for full text screening. Two authors (J.N.L./D.M. and M.B.B.) independently screened full text using our inclusion and exclusion criteria before meeting to identify included articles by consensus.

### 2.4 Framework Stage 4: Charting the Data

M.B.B. and A.D. designed and piloted a data abstraction tool based on the content of 10 articles. The tool was specifically built to work for both quantitative and qualitative studies and allowed uniform abstraction of salient points about our concept (Supporting Information S2: Appendix 2). Two researchers (A.D./J.N.L. and M.B.B.) independently abstracted data, including study date, design, population, original research question, operationalisation of social drivers and outcomes, statistics (often comparing univariate to multivariable beta coefficients) and/or quotes, and authors' conclusions. We met to discuss and resolve any differences.

### 2.5 Framework Stage 5: Collating, Summarising, and Reporting Results

Reviewers summarised their independent assessments of the 35 studies' findings and sketched ideas for the conceptual model based on the results. These included specific concepts (boxes) and links (arrows) between them, supported by the article and recorded on the data abstraction sheet. They then met to discuss their assessments and implications for the conceptual model. J.N.L. and M.B.B. collated all information from the studies into a single drafted conceptual model that was shared with all authors for final refinement.

## 3 Results

### 3.1 Study Characteristics

**FIGURE 1:**
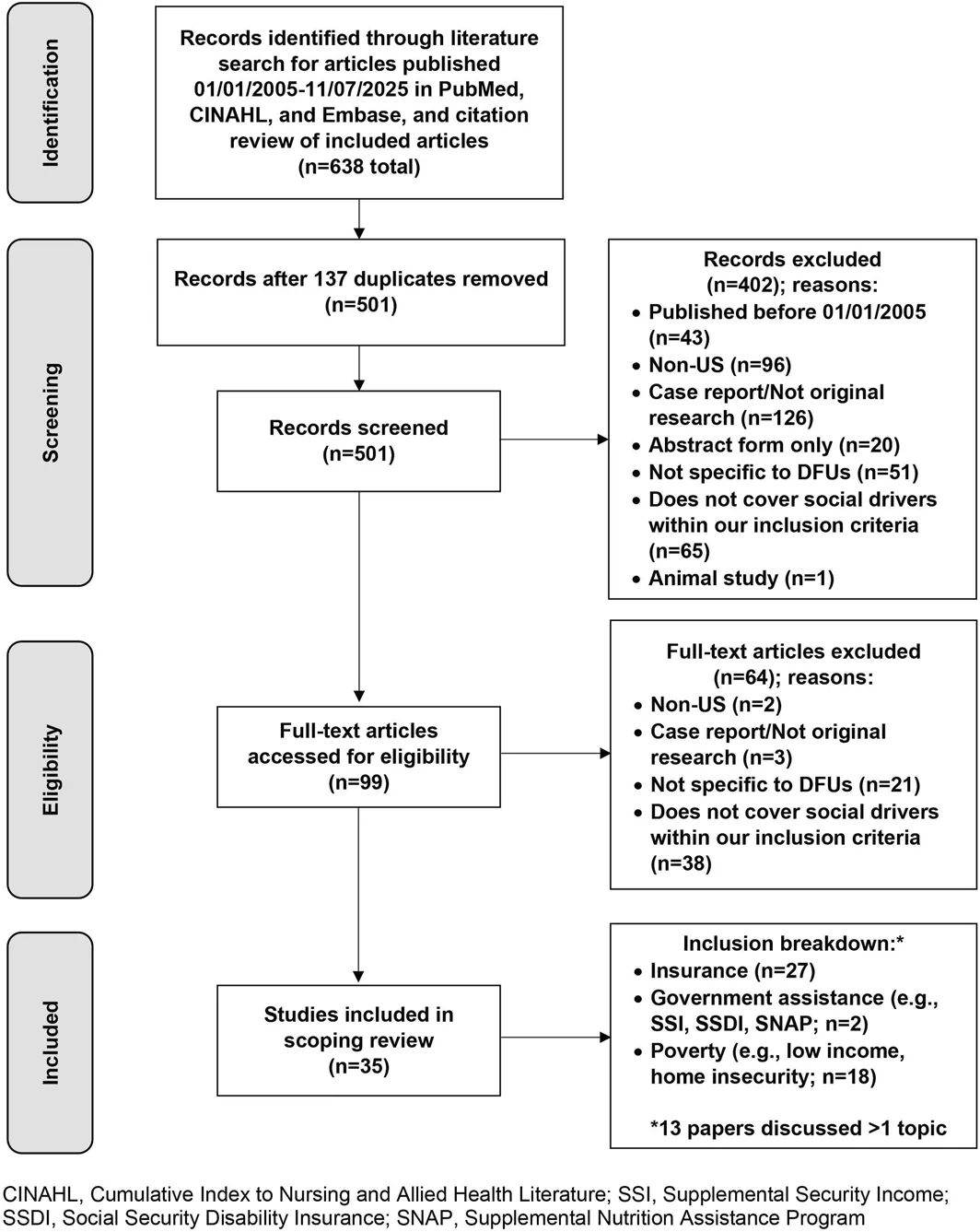
Preferred reporting items for systematic reviews and meta‐analyses (PRISMA) flow diagram.

After removing duplicates, our search identified 501 potentially eligible articles. Of these, 35 were included. The most common reasons for exclusion were as follows: case reports or non‐original research (*n* = 126), not covering one of our three social drivers (*n* = 103), occurring outside the United States (*n* = 98), and not specific to diabetic foot ulcers (*n* = 72; Figure 1).

Most of the included papers (69%) were published in or after 2020, supplying contemporary data (Table 1). Twenty‐eight (80%) studies used quantitative methods (typically a retrospective cohort design), one (3%) was a mixed methods study, and six (17%) were qualitative. Twenty‐four (69%) had a similar research question to our scoping review, although often this was to determine the magnitude of an association between a single social driver and ulcer outcome. Qualitative studies were typically more directly aligned with our aim of understanding *how* a social driver impacted outcomes. Insurance was the most studied SDDFD (*n* = 27, or 77%), with 21 (60%) studying poverty and two (6%) focussing on government assistance. Fourteen articles (40%) covered more than one SDDFD of interest. The most common outcome investigated was amputation, typically major amputation. Main and secondary points abstracted from the articles were synthesised to build a conceptual model of how poverty, insurance, and government assistance influence limb salvage for patients with diabetic foot ulcers in the United States (Table 2).

**TABLE 1:** Characteristics of 35 included studies.

Reference	Study design	Data source	Number and type of study participants	Primary outcome(s)	Social driver(s)	Was primary aim to study social drivers?
Eakers et al. [16]	Retrospective cohort	NIS	38,610 patients	Major and minor amputations	Insurance; poverty	Yes
Zhang et al. [17]	Retrospective cohort	Epic Cosmos	4,512,612 patients	Major amputation	Poverty	Yes
Kammien et al. (2025) [18]	Retrospective cohort	National PearlDiver	160,322 patients	Major and minor amputation	Insurance	Yes
Milton et al. [19]	Mixed methods (qualitative survey, interviews analysed using grounded theory)	PCPs in partnership with OPHIC	30 PCPs surveyed, seven of whom were interviewed	Major and minor amputations	Insurance; poverty	Yes
Ponukumati et al. [20]	Retrospective cohort	National Medicare	12,257,174 patients	Major amputation	Poverty	No
Samra et al. [21]	Cross sectional survey	University of Florida Clinical and Translational Science Institute’s Consent2Share database	53 patients	Financial toxicity following major amputation	Insurance; poverty	Yes
Suzuki et al. [22]	Retrospective cohort	National VHA	86,094 patients	Major amputation	Poverty	No
Khaleel et al. [23]	Retrospective cohort	Local electronic medical records	596 patients	Ulcer severity at presentation and specialty referral	Insurance; poverty	Yes
Kwon et al. [24]	Retrospective cohort	National PearlDiver	8856 patients	Major amputation	Insurance	Yes
Luu et al. [25]	Retrospective cohort	National PearlDiver	16,905 patients	Hospitalisation, major amputations, and minor amputations	Insurance	Yes
Schmidt et al. [26]	Retrospective case‐control	Local electronic medical records	22,363 patients	Ulcer complicated by infection	Poverty; government assistance	Yes
Tasman et al. [27]	Retrospective cohort	Local electronic medical records	454 patients	Ulcer severity at presentation	Poverty	Yes
Vanasse et al. [28]	Retrospective cohort	HCUP database	115,414 visit‐level encounters	Major and minor amputation	Poverty	Yes
Palmer et al. [29]	Qualitative thematic analysis	Patients from SALSA	15 patients	Ulcer outcomes and care experiences	Insurance; poverty	Yes
Crocker et al. [30]	Qualitative, phenomenologic study	Patients from SALSA	15 patients	Patient perspectives on causes of ulceration, recognition, and care‐seeking	Insurance	Yes
Fan et al. [31]	Retrospective cross‐sectional	Local electronic medical records	2009 patients	Major amputation	Poverty	Yes
Tan et al. [32]	Qualitative deductive content analysis	Patients from SALSA	15 patients	Ulcer outcomes, barriers to accessing care	Insurance; poverty	Yes
Tan et al. [33]	Retrospective Medicare cohort	State Inpatient Databases	115,071 hospitalizations	Hospitalisation, major amputation	Insurance	Yes
Crocker et al. (2021) [34]	Qualitative thematic analysis	Patients with DFU treated at a tertiary care centre	15 patients	Better understanding of patients’ lived experiences	Insurance	Yes
Flattau et al. [35]	Qualitative thematic analysis	Patients from the Bronx admitted or seen in clinic for a DFU	16 patients	Identify barriers to care	Insurance; poverty; government assistance	Yes
Zhang et al. [36]	Retrospective cohort	HSCRC database	910 patients	Re‐admission and re‐amputation (major)	Insurance; poverty	Yes
Zhang et al. [37]	Retrospective cohort	HSCRC database	7415 patients	Re‐admission and re‐amputation (minor)	Insurance; poverty	Yes
Fan et al. [38]	Retrospective cohort	NIS	31,005 hospital admissions	Major amputation	Insurance; poverty	Yes
Rhee et al. [39]	Retrospective cohort	ESRD Medical Evidence Report database	33,753 patients	Ulcer complicated by infection	Insurance; poverty	No
Hicks et al. [40]	Retrospective review of prospectively maintained database	Patients presenting to a multidisciplinary DFU service	277 patients with 621 ulcers	Ulcer healing	Insurance; poverty	Yes
Mathioudakis et al. [41]	Prospective cohort	Johns Hopkins prospective registry of MDT DFU clinic patients	217 patients with 437 ulcers	Time to ulcer healing or major amputation	Insurance	No
Jindeel et al. (2016) [42]	Retrospective cohort	California OSHPD dataset	84 hospitals with 9,794,092 discharges	Major and minor amputations	Insurance	No
Remington et al. [43]	Retrospective cohort	HCUP database supplemented with SID and SEDD	25,911 discharges	Readmission or return to emergency room	Insurance	No
Blumberg et al. [44]	Retrospective cohort	Local electronic medical records	234 patients across 3 tertiary care hospitals: 70 public hospital 103 private hospital 61 VA	Healing, major amputation, and minor amputation	Insurance	Yes
Gibson et al. [45]	Retrospective cohort	Truven Health MarketScan Research Databases	47,753 patients	Hospitalisation, major amputations, and minor amputations	Insurance; poverty	No
Rice et al. [46]	Retrospective case‐control	OptumInsight Health insurance database	32,414 matched pairs (cases and controls)	Major and minor amputation	Insurance	No
Carls et al. [47]	Retrospective cohort	Thomson Reuters MarketScan Research Databases	7528 patients	Amputation (unclear level)	Insurance	No
Margolis et al. [48]	Retrospective cohort	National Medicare	∼5 million Medicare beneficiaries with diabetes (full, national population)	Major and minor amputation	Poverty	No
Mier et al. [49]	Retrospective cohort	TIHDD records	5865 patients	Major and minor amputation	Insurance	No
Flavin et al. [50]	Retrospective case‐control	Local electronic medical records	120 patients	Infection or amputation (unclear level)	Insurance	Yes

Abbreviations: DFU, diabetic foot ulcer; ESRD, end‐stage renal disease; HCUP, Health Care and Utilization Project; HSCRC, Maryland Health Services Cost and Review Commission; MDT, multi‐disciplinary team; NIS, National Inpatient Sample; OPHIC, Oklahoma Primary Healthcare Improvement Cooperative; OSHPD, Office of Statewide Health Planning and Development; PCPs, Primary Care Providers; SALSA, Southwestern Academic Limb Salvage Alliance; SEDD, State Emergency Department Database; SID, State Inpatient Databases; TIHDD, Texas Inpatient Hospital Discharge Data; VHA, Veterans Health Administration.

**TABLE 2:** The main points of the 35 included articles, organised by social driver.

Social driver	Article	Main point(s)
Insurance	Eakers et al. [16]	Patients without insurance were more likely to have infectious complications and minor amputations. Patients without insurance were more likely to be discharged to home instead of to a facility.
	Kammien et al. [18]	Compared to those with private insurance, Medicare and Medicaid beneficiaries were less likely to undergo limb salvage procedures. However, if Medicare and Medicaid beneficiaries had limb salvage procedures, they were only slightly less likely to require an amputation compared with privately insured patients.
	Khaleel et al. [23]	Specialty care referral source (e.g., primary care or endocrinology vs. emergency or self‐referral) did not vary based on insurance type.
	Kwon et al. [24]	Patients who switched from Medicaid to commercial insurance had a lower risk of major amputation compared to those who remained on Medicaid.
	Luu et al. [25]	Compared to Medicaid patients residing in a state where their insurance did *not* cover podiatric services, Medicaid patients residing in a state where their insurance covered podiatric services were (1) less likely to be hospitalised or undergo a major amputation and (2) more likely to undergo minor amputations.
	Crocker et al. [30]	Suboptimal insurance coverage contributed to, but was probably not the main driver of, late patient presentation after developing an ulcer. Suboptimal insurance posed barriers to accessing care, especially specialists and shoe gear.
	Tan et al. [32]	Even when patients living in poverty had insurance, there was still significant financial strains (e.g., cost of orthotics and dressing supplies) that impacted care choices.
	Tan et al. [33]	Early adoption of the Affordable Care Act was associated with significant decreases in major amputations and hospitalizations, especially among previously uninsured patients.
	Crocker et al. [34]	Subpar insurance exacerbated financial hardships due to mounting medical bills. Amputations/off‐loading restrictions led to a feedback loop of job loss, less robust insurance and less income, and increased risk of subsequent ulcerations.
	Flattau et al. [35]	Among a group of disenfranchised patients with diabetic foot ulcers (75% on SSDI, 6% with private insurance, and 31% living in a home that was too crowded), patients reported limited understanding of ulcer risk, late recognition of cues to seek medical attention, impaired access to healthcare, encountered social needs as barriers to care, and experienced their first ulcers as a ‘wake‐up call’ for improving their self‐care.
	Zhang et al. [36]	Among patients who underwent major amputation, those with Medicare insurance, compared to private, had higher rates of 30‐day re‐hospitalisation but not re‐amputation.
	Zhang et al. [37]	Among patients who underwent minor amputations, those with Medicare or Medicaid insurance, compared to private, had higher rates of 30‐day re‐hospitalisation. This risk was attenuated by about half after controlling for other demographics and comorbidities. Among patients who underwent minor amputations, those with Medicare or Medicaid insurance, compared to private, had higher rates of re‐amputation. This risk was attenuated by about half after controlling for other demographics and comorbidities.
	Fan et al. [38]	Patients with private insurance, compared to those without, were more likely to undergo limb salvage procedures and less likely to undergo amputation. Controlling for comorbidities significantly attenuated this relationship, suggesting the effect of private insurance on limb salvage was strongly mediated by comorbidities.
	Rhee et al. [39]	Dialysis patients who were Medicare/Medicaid dual eligible were more likely to have higher A1Cs than those with Medicare alone, and dialysis patients with higher A1Cs were more likely to develop a diabetic foot infection, independent of comorbidities, lab values, and vascular access.
	Hicks et al. [40]	Among patients treated in an academic, multidisciplinary limb salvage centre, Medicaid insurance, compared to Medicare, was associated with improved wound healing.
	Mathioudakis et al. [41]	Patients with Medicare or Medicaid insurance, compared to private, were more likely to present with more severe ulcers. Among patients who progressed to amputation, infection was often the tipping point.
	Jindeel et al. [42]	Californian municipal hospitals, compared to non‐profit or private hospitals, admitted more Medi‐Cal patients and performed more amputations, but the majority of these were minor amputations. Non‐profit and private, compared to municipal, hospitals admitted more private and Medicare insured patients. Medicare patients in these hospitals, compared to municipal hospitals, were more likely to undergo amputation. Although fewer amputations were performed in non‐profit and private hospitals, compared to municipal hospitals, the proportion of major amputations was higher.
	Remington et al. [43]	Patients with Medicare or Medicaid insurance, compared to private, were more likely to be readmitted, even after controlling for aggressiveness of care during the index hospitalisation, length of stay, comorbidities, and other patient demographics.
	Blumberg et al. [44]	Having VA insurance, compared to private, Medicaid, or Medicare, was associated with non‐healing ulcers in a model that controlled for ulcer‐ but not patient‐level factors.
	Gibson et al. [45]	Regardless of insurance type, patients who saw a podiatrist before an ulcer developed were much less likely to require amputation or hospitalisation when an ulcer did occur.
	Rice et al. [46]	Patients with Medicare insurance, compared to private, had a lower unadjusted risk of amputation, receiving advanced therapies, and hospitalizations.
	Carls et al. [47]	Patients with Medicare and supplemental commercial insurance, compared to commercial, were less likely to undergo amputation. In both insurance groups, podiatric care prior to ulceration was protective against amputation. However, prior podiatric care benefited those with commercial insurance more than those with Medicare and supplemental commercial insurance.
	Mier et al. [49]	Patients with Medicare or Medicaid, compared to employer‐based insurance, were more likely to undergo amputation.
	Flavin et al. [50]	Patients without insurance, compared to those with insurance other than Medicaid, were more likely to be admitted with infected ulcers and undergo amputation.
Poverty	Eakers et al. [16]	Poverty remained associated with amputation after controlling for insurance.
	Zhang et al. [17]	A lack of housing is the facet of poverty that is most strongly associated with poor outcomes.
	Milton et al. [19]	Poorer patients are more likely to be served by clinics that are structurally disempowered, lacking resources to rapidly refer to outpatient limb salvage specialists. This leads to crisis care in emergency rooms and late presentations, which begets amputations.
	Ponukumati et al. [20]	Poverty was associated with minor amputations, which then drove up the risk of subsequent, major amputation.
	Samra et al. [21]	Major amputations worsen financial hardships, with most amputees transitioning to unwelcome unemployment. Financial hardships triggered by major amputation are independent of insurance.
	Suzuki et al. [22]	The association between the social vulnerability index and amputation was significantly dampened by controlling for things along the posited causal pathway, including which hospital provided care, comorbidities, and ulcer severity. Poverty, measured by the social vulnerability index, remained associated with amputation after controlling for insurance.
	Khaleel et al. [23]	Specialty care referral source (e.g., primary care or endocrinology vs. emergency or self‐referral) did not vary based on area deprivation index.
	Schmidt et al. [26]	Patients living in less affluent neighborhoods were more likely to present with infected ulcers.
	Tasman et al. [27]	Patients living in distressed neighborhoods were more likely to present with severe ulcers, especially ulcers complicated by more extreme ischaemia and infection.
	Vanasse et al. [28]	Access to, and use of, public transportation was associated with limb salvage for patients living in affluent neighborhoods but not low‐income neighborhoods.
	Palmer et al. [29]	Low paying, high labour jobs meant patients were more likely to get an ulcer. Once an ulcer developed, patients had to choose between not working to adhere to off‐loading recommendations and exacerbating financial hardships with increased risk of amputation, or working with subpar off‐loading compliance which also increased the risk of amputation.
	Fan et al. [31]	The area deprivation index remained independently associated with major amputation after controlling for other sociodemographics and comorbidities within a healthcare system with minimal insurance constraints (Veterans Administration).
	Tan et al. [32]	Patients living in poverty had significant health literacy gaps that impeded their ability to make informed decisions about when and how to seek care. Patients living in poverty tended to get information about ulcer prevention after, not before, their first ulcer. Patients living in poverty tended to have fragmented relationships with their clinicians.
	Flattau et al. [35]	Among a group of disenfranchised patients with diabetic foot ulcers (75% on SSDI, 6% with private insurance, and 31% living in a home that was too crowded), patients reported limited understanding of ulcer risk, late recognition of cues to seek medical attention, impaired access to healthcare, encountered social needs as barriers to care, and experienced their first ulcers as a ‘wake‐up call’ for improving their self‐care.
	Zhang et al. [36]	Among patients who underwent a major amputation, living in a disadvantaged neighbourhood was associated with a higher rate of re‐amputation but not 30‐day re‐hospitalisation.
	Zhang et al. [37]	Among patients who underwent minor amputations, living in a disadvantaged neighbourhood was associated with a higher rate of 30‐day re‐hospitalisation. This risk was attenuated by about half after controlling for other demographics and comorbidities. Among patients who underwent minor amputations, living in a disadvantaged neighbourhood was associated with a higher risk of re‐amputation. This risk was attenuated by about half after controlling for other demographics, comorbidities, and re‐hospitalisation.
	Fan et al. [38]	Patients living in neighborhoods with lower median household incomes were more likely to undergo amputation rather than limb salvage. Hospital‐level factors (e.g., access to an urban teaching hospital) more strongly attenuated living in neighborhoods with lower median household incomes than insurance. Access to urban teaching hospitals, where limb salvage teams are housed, was the most protective factor against amputation.
	Rhee et al. [39]	Dialysis patients living in neighborhoods with lower median household incomes and higher proportions of residents living below the poverty line were more likely to have higher A1Cs, and dialysis patients with higher A1Cs were more likely to develop a diabetic foot infection, independent of comorbidities, lab values, and vascular access.
	Hicks et al. [40]	Among patients treated in an academic, multidisciplinary limb salvage centre, area deprivation index was not associated with worse wound healing outcomes.
	Gibson et al. [45]	Regardless of insurance, patients who lived in neighborhoods with lower median household incomes were less likely to see a podiatrist before a diabetic foot ulcer developed, care which protected against hospitalisation and amputation when an ulcer did occur.
	Margolis et al. [48]	Amputations increased as regional socioeconomic status decreased, an association that was partially blunted by controlling for diabetes, vascular disease, ulceration, age, sex, race, and prevalence of clinicians.
Government assistance	Schmidt et al. [26]	Patients living in neighborhoods with a higher proportion of residents receiving public assistance income or food stamps were more likely to present with infected ulcers.
	Flattau et al. [35]	Among a group of disenfranchised patients with diabetic foot ulcers (75% on SSDI, 6% with private insurance, and 31% living in a home that was too crowded), patients reported limited understanding of ulcer risk, late recognition of cues to seek medical attention, impaired access to healthcare, encountered social needs as barriers to care, and experienced their first ulcers as a ‘wake‐up call’ for improving their self‐care.

### 3.2 Conceptual Model Overview: Placing Poverty, Insurance, and Government Assistance on a Path to Ulcer Outcomes

**FIGURE 2:**
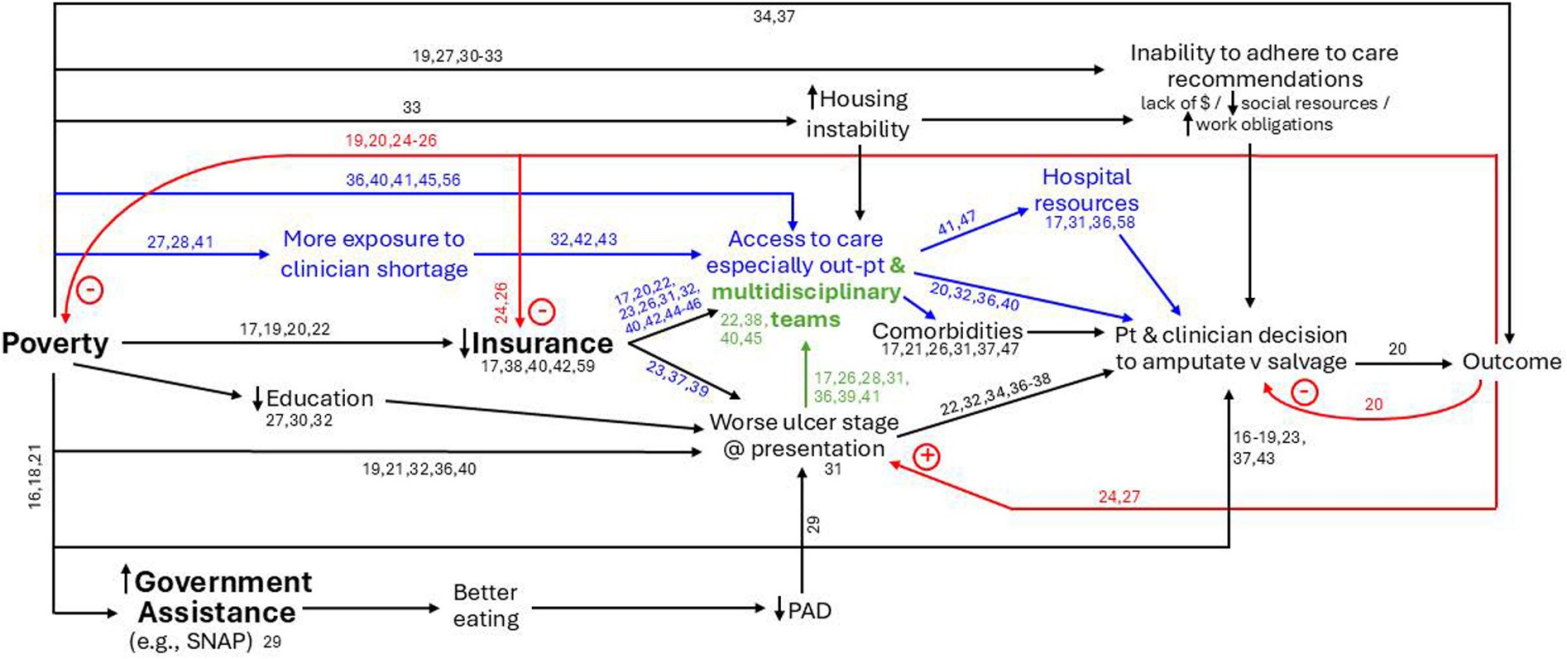
Conceptual model of how poverty, insurance, and government assistance impact diabetic foot ulcer outcomes. Numbers correspond to studies included in our scoping review. Blue indicates pathways involving the healthcare system. Green represents the potential of multidisciplinary teams to favourably mediate the influence of poverty and insurance on limb salvage. Red represents feedback loops from the outcome to points along the posited causal pathway. We included directionality when this was clear and consistent (e.g., poverty was associated with *lower* education or *inability* to comply with care recommendations). We did not include directionality when data were conflicting or, more commonly, directionality depended on the antecedent variable (e.g., ulcer stage at presentation would be less severe if following the pathway through lower peripheral vascular disease but more severe if coming from the lower education pathway).

How social drivers, especially poverty, impact diabetic foot ulcer outcomes is complex. Our conceptual model proposes multiple paths originating from poverty (Figure 2). Insurance and government assistance are more proximal to ulcer outcomes, and each was on separate lines stemming from poverty [20, 29, 33, 38, 39, 44, 49].

We placed insurance on a prominent pathway between poverty and ulcer outcomes because low income is often a prerequisite for Medicaid. Furthermore, many patients in financial hardship are uninsured or underinsured. Fan and colleagues demonstrated that the association between insurance and major amputation was substantially attenuated after adding comorbidities into the model, suggesting that comorbidities mediate much of the effect of subpar insurance on ulcer outcomes. In contrast, the association between income and major amputation was not substantially changed when comorbidities were added, suggesting that poverty impacts outcomes through pathways other than comorbidities alone [38]. Another study focused on treatment; compared to those with commercial insurance, patients with Medicaid or Medicare were less likely to undergo limb salvage procedures and more likely to undergo major amputation [18]. While the association between subpar insurance and poor outcomes is well described, our scoping review also highlighted a seldom recognized feedback loop between poor ulcer outcomes, especially amputations, and both poverty and insurance [20, 21, 29, 34, 36]. Specifically, patients living in poverty and working physically demanding jobs (e.g., construction, waiting tables) often faced a harrowing dilemma: if they complied with off‐loading recommendations, they frequently risked unemployment, but if they continued working physically demanding jobs, their ulcers often progressed to require amputation [29, 34]. Either scenario led to a vicious cycle of further poverty, loss of any employer‐sponsored insurance, increased risk of recurrent ulceration, and poor outcomes [21, 29, 34].

We placed government assistance as an intermediary between poverty and foot ulcer outcomes because low income is often a prerequisite for government assistance. The two articles that reported on government assistance used specific programmes as a marker of poverty, rather than a way to understand how government assistance programmes may impact ulcer outcomes [26, 35]. When doing so, the authors correctly interpreted their findings as poverty's association with poor outcomes rather than the specific government assistance causing poor outcomes. One study incorporated public assistance income and/or Supplemental Nutrition Assistance Programme (SNAP) into a composite poverty index, associating this index with the risk of developing infection [26]. The other was a qualitative study of patients with diabetic foot ulcers, 75% of whom received Social Security Disability Insurance (SSDI). Study participants described limited understanding of ulcer risk, late recognition of cues to presentations, impaired access to healthcare, and high social needs that created barriers to treatment [35]. This study also highlighted a constructive feedback loop, where a first ulceration served as a ‘wake up call’—patients reported improved knowledge and earlier presentation when ulcers recurred. Only one study, which measured economic distress across Appalachian counties, hypothesised how a government assistance programme may positively impact diabetic foot ulcer outcomes. They reasoned that SNAP should promote better diets, leading to reduced vascular disease, and fewer ulcers complicated by ischaemia [27]. However, at the time of publication, the authors had no direct evidence to support their hypothesis.

Poverty also impacts outcomes through paths independent of insurance and government assistance. Low levels of education, housing instability, and the inability to adhere to care recommendations were repeatedly found to stem from poverty and lead to poor outcomes [17, 29, 30, 32, 35, 40]. One study indicated that being unhoused was the single most important social factor determining ulcer outcomes, significantly impeding access to high‐quality care and adherence to care recommendations [17]. Another study within the Veterans Administration system found that the association between poverty and amputation was minimally attenuated after controlling for comorbidities, suggesting that decisions to forgo limb salvage for amputation were driven by social barriers to treatment more than disease or insurance‐driven barriers to care [31]. The authors of this study speculated that these social barriers may include limited transportation, stress, and poor social support [31]. Other researchers found evidence to support reasons why patients were unable to adhere to recommendations needed for limb salvage, including lack of money (e.g., to buy bandages or afford to take time off from work), increased work obligations (e.g., inability to off‐load on labour intensive jobs), and decreased social supports (e.g., no one available to help with household chores, wound care, or transportation to clinic appointments) [29, 37, 38, 51]. However, these factors constitute an incomplete list of SDDFD along the pathway between poverty and limb salvage, evidenced by a continued association between the two after controlling for a myriad of variables [16, 31].

### 3.3 Late Presentation to Care

Poverty was linked to late presentations and more severe ulcers at the time of diagnosis, making treatment more difficult and directly influencing the decision to pursue amputation over limb salvage [16, 32, 33, 37, 43]. Minimal health literacy and less formal education were correlated with limited knowledge about diabetic foot ulcers and a consistent reason why patients living in poverty tended to present late with severe ulcerations [18, 30, 32, 35, 43]. One study of patients living in poverty found that they typically did not receive foot care education, including ulcer prevention and warning signs, until after developing an ulcer [32]. A qualitative study described a positive feedback loop, wherein patients with recurrent ulcerations learned through experience to present earlier for care [35]. However, this positive result was insufficient to overcome the negative impact of poverty on ulcer outcomes.

In addition to minimal food care education, subpar insurance factored into why patients living in poverty presented late with more severe ulcerations [16, 18, 41]. Those without insurance—compared to Medicaid, Medicare, or commercial insurance—were more likely to present with late‐stage, infected ulcers [16]. Another study noted that as ulcer severity at presentation increased, so did the proportion of patients insured through Medicare or Medicaid, compared with commercial carriers [41]. One also noted the counterpoint: patients whose Medicaid insurance covered podiatric services were more likely to seek care at earlier stages, including preventative services [25].

### 3.4 Healthcare System Factors

Healthcare systems exist within a broader social context. Multiple studies have provided evidence regarding how poverty impacts (1) the type of medical care patients with diabetic foot ulcers receive, and, consequently, (2) ulcer outcomes. Patients living in poverty also tended to live in areas with clinician shortages, resulting in longer drive times, wait times, last‐minute cancellations, and less frequent follow‐ups [19, 26, 35]. Some patients living in poverty were minimally skilled at self‐advocacy and described fragmented relationships with healthcare providers [32, 35]. Both facets negatively impacted preventive care and ulcer treatment. However, the net provision of more care—as measured by Medicare expenditures—did not necessarily correlate with reductions in amputations. This suggests that simply expanding access is desirable but insufficient to improve outcomes; patients need ready access to the right clinicians and therapies for the problem at hand, rather than indiscriminate resources [24, 25, 33, 43, 48].

Multiple studies supported the association between insurance and access to care, especially outpatient and multidisciplinary care [18, 24, 25, 32, 33, 40, 45, 46, 47]. When patients' insurance covered specialty limb care, they tended to seek care in outpatient settings, a behaviour associated with decreased risks of hospitalisation and major amputation [25, 45, 47]. Two studies in particular suggested that access to specialty care could partially compensate for the negative impact of poverty on ulcer outcomes. A single‐centre study of patients receiving care at an outpatient multidisciplinary clinic reported no difference in amputations based on poverty [40]. Kammien and colleagues found that patients with Medicare and Medicaid, compared with those with commercial insurance, were less likely to undergo limb salvage procedures [18]. However, if these patients *did* undergo advanced limb salvage surgery, their outcomes were on par with their commercially insured peers. While provocative, both studies may be limited by residual confounding; patients living in poverty who are able to navigate to these teams may have more social resources and fewer comorbidities than their counterparts. We also found evidence to support a pattern whereby patients with low income and subpar insurance presented to emergency rooms with very severe ulcers that required admission [19, 38, 40, 41]. For those hospitalised at large tertiary care centres, this often precipitated rapid access to advanced limb salvage teams [38, 41]. Expedited access from the time of presentation for some of the most disadvantaged patients seems paradoxical, but it can be explained by emergency room and hospital utilization [40]. In these instances, expedited access did not necessarily improve outcomes due to late‐stage ulcer presentations at the time of presentation [19].

The type of hospital that provided care was a very strong predictor of ulcer outcomes and closely tied to socioeconomic status [24, 25, 33, 43, 48]. In one study, admission to a tertiary teaching hospital was the strongest predictor of limb salvage [38]. Even within the Veterans Affairs system, relatively insulated from the effects of insurance, facility‐level variation in amputation rates persisted [22]. In another study, only about half of the association between poverty and ulcer outcomes could be explained by patient‐level factors, including demographics and comorbidities, suggesting that healthcare system factors heavily influence patient outcomes [37]. To understand this better, Milton and colleagues conducted a study of clinicians practicing in Oklahoma and serving disadvantaged patients, including rural clinics, free clinics, and clinicians with > 50% of their patient panels receiving Medicare or lacking insurance [19]. They described ‘structural disempowerment’, whereby the clinics serving patients most in need were also the least resourced and least connected to tertiary care centres of excellence: clinicians reported this lack of support drove patients to seek crisis care in the emergency room when ulcers were at very late stages and limb salvage was unlikely.

Evidence suggests that healthcare system resources impacted patient‐clinician decisions regarding amputation. In states whose Medicaid programmes covered podiatric care, there was a shift away from hospitalizations and major amputations but a concurrent shift towards minor amputations (perhaps as a means of avoiding major amputations) [25]. Zhang and colleagues report that patients who underwent minor amputations at hospitals with higher ulcer‐specific readmission rates—a potential marker for under‐resourced systems—had higher 1‐year re‐amputation rates even after controlling for patient sociodemographics and comorbidities [37]. This pattern was echoed in a study where patients living in poorer neighborhoods were more likely to progress to major amputations after undergoing a minor amputation [20].

## 4 Discussion

We present a conceptual model of how three social drivers—poverty, insurance, and government assistance—impact diabetic foot ulcer outcomes (Figure 2). Our model underscores the complexity of this problem and emphasises the interface between healthcare systems and social drivers. Our intent was to build a model that could inform future regression analyses by better understanding how change in one variable impacts other variables in the causal pathway and, ultimately, ulcer outcomes. We conclude by discussing the implications for this use.

First, our conceptual model is not a directed acyclic graph. It contains feedback loops between the outcome and variables along the causal pathway. When used to inform regression analysis, we suggest restricting to patients with incident ulcers to avoid the impact of these feedback loops. While ulcers can be longstanding, a rational approach would be to require a 1‐ or 2‐year ulcer‐free period before an index diagnosis [52]. If this is not feasible, it may be most important to limit it to those without a prior major amputation because this outcome arguably has the largest impact on subsequent poverty and insurance [21, 34]. The feedback loop between outcome and poverty is an important reminder that limb salvage has a social benefit. While preventing amputation will never cure poverty, it makes a meaningful contribution to the social well‐being of our patients.

Our conceptual model is rife with mediators, especially within the healthcare system. Mediators are on the causal pathway between SDDFD and ulcer outcomes [53, 54]. They partially account for the effect of a SDDFD on outcomes (Table 3). Our model posits that healthcare mediators of poverty's effect on ulcer outcomes include clinician shortages, access to care, and hospital resources. Researchers wishing to fully account for the effect of poverty on ulcer outcomes should exclude these healthcare factors in their statistical models to avoid underestimating the overall impact of poverty [56]. However, if the intent is to estimate the extent to which a healthcare system variable may partially compensate for poverty, then formal mediation analysis may prove useful. The joint significance test of alpha and beta is one example of a statistical approach to assess for mediation, and it would be a reasonable next step to investigate the mediation pathways depicted in our model [53, 54].

**TABLE 3:** Mediator, moderator, and collider definitions and examples.

Term	Definition	Directed acyclic graph representation [55]	Statistical modelling	Non‐scientific example	Diabetic foot ulcer example
Mediator [53, 54, 56]	A variable functions as a mediator to the extent that it accounts for the effect of a predictor on an outcome; mediators speak to how or why such effects occur.	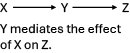	Joint significance test of *α* and *β*	The total effect of practice (X) on performance (Z) is mediated by how much a saxophonist's fingering improves (Y). If the musician practices a lot, and their fingering does not improve, performance might not either.	The effect of poverty (X) on ulcer outcomes (Z) is mediated by access to care (Y).
Moderator [53]	A variable functions as a moderator to the extent that it affects the direction or magnitude of a relationship between a predictor and outcome; moderators partition predictors into subgroups so that each subgroup can have different relationships with the outcome.	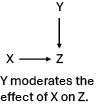	Interaction term of X and Y	Playing a high‐quality saxophone (Y) moderates the effect of a musician's training level (X) on his/her performance (Z); it will benefit the performance of professional musicians more than beginner musicians.	An inability to adhere to recommendations (Y) moderates the effect of insurance (X) on ulcer outcomes (Z); it will likely impact those with subpar insurance more than those with robust coverage.
Collider [57, 58]	A variable is termed a collider when: (a) It is directly affected by two or more other variables, (b) those variables are independent of one another, and (c) the colliders and other variables are associated with the outcome.	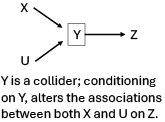	Conditioning (stratification or regression adjustment) on a collider will alter the estimated association between the antecedent variables and the outcome, often resulting in collider bias. Excluding the collider is likely to yield more valid causal inference.	Practice (Y) is a collider with respect to teaching (X) and talent (U). Teaching and talent are independent of each other, at least in the beginning of a musician's training. Practice is directly affected by teaching because great teaching inspires practice. Innate talent also spurs practice because it is enjoyable to play. Teaching, talent, and practice all impact performance (Z). However, if we condition on practicing, we may alter the association between (a) talent and performance, and (b) teaching and performance. We might falsely conclude that musicians who are less talented perform better, or those with poorer teachers perform better because we are already selecting for musicians who practice a lot.	Access to care (Y) is a collider with respect to clinician shortage (X) and insurance (U). Clinician supply and insurance are independent. Access to care is directly affected by clinician supply and insurance. All three are associated with ulcer outcomes (Z). Conditioning on access may result in falsely concluding that clinician shortage or subpar insurance is associated with improved outcomes because we are already selecting for patients with access to care.

Our model suggests that insurance mediates poverty but does so incompletely. This has important implications for researchers who use insurance status as a proxy for poverty. Specifically, when researchers substitute Medicaid insurance or uninsured status for poverty, our model suggests that they are underestimating the impact of poverty on ulcer outcomes. We think underestimations are likely to occur because our model includes facets—such as clinician shortages—that are on the causal pathway between poverty and outcome, but not on the pathway between insurance and outcome [19, 26, 35]. Furthermore, while we placed access to care on the causal pathway between insurance and outcome, we found evidence that poverty also impacts access to care in ways that are independent of insurance. Our model suggests that when the intent is to measure the full effect of poverty on ulcer outcomes, insurance should not be included [56]. Alternatively, controlling for insurance would yield an estimate of the effect of poverty on outcomes independent of insurance. This has important implications for policies such as Medicare expansion because it estimates the remaining deficit due to poverty if insurance was equalised over the population.

Inability to adhere to care recommendations is a prominent moderator of other facets along the causal pathway between poverty and ulcer outcomes in our model [17, 29, 30, 32, 35, 40]. A moderator differentially impacts subgroups of a variable along the causal pathway, so that each subgroup can have different associations with the outcome (Table 3) [53, 54]. Here, an inability to adhere to care recommendations may explain why some patients with robust access to care and resource‐rich hospitals still experience poor outcomes. The opposite may also be true: a strong ability to adhere to care recommendations may explain why some patients with poor access to care and resource‐strapped hospitals are able to have good outcomes. Similarly, our model suggests that outcomes may differ among patients with the same insurance, in part due to differences in their ability to adhere to care recommendations. The ability to adhere may moderate the association between insurance and outcomes. Unfortunately, measuring the ability to adhere is difficult and simply missing in large claims databases. New survey tools are being developed to fill this gap [59]. If researchers succeed in quantifying this concept, stratification analysis or including an interaction term between the ability to adhere and other facets along the causal pathway would be ways to assess for these moderating relationships.

Researchers may want to use our model to minimise potential collider‐conditioning bias in their regression models, which can be insidious without a causal diagram [57]. Colliders are variables where two or more causal pathways converge (i.e., two or more arrowheads point towards a single variable; Table 3) [57]. Conditioning on a collider, through restricting, stratification, or regression adjustment, can result in spurious, often paradoxical, statistical associations [58]. In our model, the most overt collider is the patient and clinician decision to pursue amputation or limb salvage; six arrowheads converge here. However, this is unlikely to result in collider‐conditioning bias because decision‐making is seldom measured or included in statistical models. Our model also contains fewer overt, putative colliders. For instance, access to care may act as a collider with respect to clinician shortages and insurance [18, 20, 24, 25, 32, 33, 36, 38, 40, 45, 46, 47, 48, 51]. If researchers were to include access as a covariate, collider‐conditioning may result in areas with clinician shortages being associated with lower amputation risks or patients with subpar insurance having lower estimated risk of amputation than those with full coverage. This is because the model already accounts for access to care, a downstream event to clinician shortage and insurance.

Our model suggests that the healthcare system is a promising way to partially compensate for the impact of SDDFD on ulcer outcomes. In particular, multidisciplinary teams based in tertiary‐care settings may mitigate the negative effects of poverty and lacklustre insurance [38, 40]. However, equitable access remains critical, especially with respect to outpatient services. This includes the role of triage across rural and community‐based clinics into academic centres [19, 22, 38, 40, 45]. Care patterns, such as crisis care in emergency rooms, should be recognized. Interventions aimed at improving access to outpatient multidisciplinary services may want to not only track intake into these clinics, but also potential reductions in emergency room use, hospitalizations, and lengths of stay. The model also has broader, policy‐based implications for further subsidising clinics serving the underprivileged to combat ‘structural disempowerment’ in the healthcare system itself [19].

Like all models, ours is imperfect. Statistical regressions exploring putative mediators, moderators, and colliders could generate data substantiating or refuting our proposed associations (Table 3). Furthermore, we need to begin measuring what is most impactful, not what is most convenient. Qualitative studies identified some of these key variables, such as an inability to adhere to care recommendations (and why) as well as patient‐prioritised outcomes (perhaps function rather than amputation) [19, 29, 30, 32, 34, 35, 60]. Quantitative methods are needed to measure and adequately model these variables—methodological approaches that will likely involve surveys of prospective cohorts, such as the Diabetic Foot Consortium's. However, we need to remain cognizant that patients enroled in these cohorts are typically served at pinnacle multidisciplinary clinics and able to follow study protocols, which might be a surrogate for their ability to adhere to care recommendations. Results may not generalise to the national population with diabetic foot ulcers. Finally, our model was derived from limited data. Our scoping review contained scarce information on how government assistance programmes, such as SNAP, may impact diabetic foot ulcer outcomes. Studies also demonstrated a persistent association between poverty and poor outcomes even after controlling for variables included in our model [16, 31]. This suggests that further, unrecognised pathways exist.

While our model of how poverty, insurance, and government assistance may impact diabetic foot ulcers is imperfect, we think it may be useful. In particular, the model suggests roles for mediators, moderators, and colliders in this complex causal pathway. These roles can be assessed with thoughtful statistical modelling. We think that they can be used to identify intervention points, especially in the healthcare system, that can impact health disparities rooted in social drivers.

## Author Contributions

J.N.L. assisted with the scoping review design, collected and analysed data, contributed to the first draft, and reviewed the final manuscript. D.M. and A.D. collected and analysed data and reviewed the final manuscript. M.S.C., M.F., A.O., M.C.S., and J.C.I. contributed to the overall study design, finessed analysis, and reviewed the final manuscript. C.S. and W.Y. assisted in interpreting the statistical implications of the emerging conceptual model and reviewed the final manuscript. M.B.B. contributed to the overall study design, scoping review protocol, and data analysis. She wrote the first draft and reviewed the final draft.

## Funding

This study was conducted by the NIH‐sponsored Diabetic Foot Consortium supported through Grant IDs U01DK119100, U01DK119083, U01DK119094, U01DK119099, and U24DK122927 of the National Institute of Diabetes and Digestive and Kidney Diseases (NIDDK).

## Conflicts of Interest

The authors declare no conflicts of interest.

## Supporting information


Supporting Information S1

Supporting Information S2

## Data Availability

Data sharing not applicable to this article as no datasets were generated or analysed during the current study.
