# Supplementary material for: How Do Poverty, Insurance, and Government Assistance Impact Diabetic Foot Ulcer Outcomes? A Scoping Review
Source: Diabetes Metab Res Rev. 2026 Aug 21;42(6):e70219. doi: 10.1002/dmrr.70219 (PMC13495135; doi:10.1002/dmrr.70219)
Supplement: Supplementary file 1 [file DMRR-42-e70219-s001.docx]

**SUPPLEMENTARY MATERIALS**

This appendix has been provided by the authors to give readers additional information about their work.

Supplement to: LaMantia JN, McClain D, Brennan MB, et al. How do poverty, insurance, and government assistance impact diabetic foot ulcer outcomes? A scoping review. 2026.

**APPENDIX 2. Data Abstraction Tool**

| **Scoping Review Details** | |
| --- | --- |
| Scoping Review Title | How poverty, government assistance, and insurance status may impact diabetic foot ulcer care and outcomes: a scoping review |
| Review Objective(s) | We know that social drivers of health impact diabetic foot ulcer outcomes. This scoping review aims to understand potential mechanisms behind these impacts. The ultimate goal is to compile a comprehensive conceptual model of how social drivers impact foot ulcer outcomes and inform future mediation analysis. |
| Review Questions | How does 1) poverty, 2) government assistance, and 3) insurance status impact diabetic foot ulcer care processes or outcomes? |
| **Article Details** | |
| Manuscript Title |  |
| Citation (YEAR- Last name of 1^st^ author) |  |
| PMID |  |
| Reference’s Concept/Study Aim |  |
| Study type (e.g. retrospective cohort) |  |
| Population (data source and dates) |  |
| Participants (numbers of participants/ unit of analysis) |  |
| **Inclusion/Exclusion Criteria** | |
| Reference inclusion criteria |  |
| Reference exclusion criteria |  |
| **Outcomes and Social Drivers of Health** | |
| Outcome |  |
| Operationalization of outcome |  |
| Social driver(s) of health:  Insurance  Government assistance  Poverty |  |
| Operationalization of social driver(s) of health |  |
| **Details/Results Extracted from Source of Evidence** | |
| Take home findings |  |
| Statistics or quotes to back up the take home findings |  |
| Authors’ thoughts about why social drivers of health might impact care processes and ulcer outcomes |  |
| Our thoughts about why |  |
| Implications for conceptual model |  |
