# Supplementary material for: How Do Poverty, Insurance, and Government Assistance Impact Diabetic Foot Ulcer Outcomes? A Scoping Review
Source: Diabetes Metab Res Rev. 2026 Aug 21;42(6):e70219. doi: 10.1002/dmrr.70219 (PMC13495135; doi:10.1002/dmrr.70219)
Supplement: Supplementary file 2 [file DMRR-42-e70219-s002.docx]

**SUPPLEMENTARY MATERIALS**

This appendix has been provided by the authors to give readers additional information about their work.

Supplement to: LaMantia JN, McClain D, Brennan MB, et al. How do poverty, insurance, and government assistance impact diabetic foot ulcer outcomes? A scoping review. 2026.

**APPENDIX 1. Search Terms**

**PUBMED SEARCHES**

**MeSH term search:** ((foot ulcer, diabetic [MeSH Terms]) AND (united states[MeSH Terms]) AND ((social determinants of health[MeSH Terms]) OR (medicaid[MeSH Terms]) OR (medicare[MeSH Terms]) OR (medically uninsured[MeSH Terms]) OR (food assistance[MeSH Terms]) OR (food insecurity[MeSH Terms]) OR (ill-housed persons[MeSH Terms]) OR (public housing[MeSH Terms]) OR (housing instability[MeSH Terms]) OR (social welfare[MeSH Terms]) OR (unemployment[MeSH Terms]) OR (poverty[MeSH Terms]) OR (socioeconomic factors[MeSH Terms])))

**General search:** (diabetic foot ulcer) AND (united states) AND ((social determinants of health) OR (medicaid) OR (medicare) OR (medically uninsured) OR (food assistance) OR (food insecurity) OR (ill-housed persons) OR (public housing) OR (housing instability) OR (social welfare) OR (unemployment) OR (poverty) OR (socioeconomic factors))

**CINAHL SEARCH**

"diabetic foot ulcer" AND "united states" AND ("social determinants of health" OR "medicaid" OR "medicare" OR "medically uninsured" OR "food assistance" OR "food insecurity" OR "ill-housed persons" OR "public housing" OR "housing instability" OR "social welfare" OR "unemployment" OR "poverty" OR "socioeconomic factors")

**EMBASE SEARCH**

'diabetic foot ulcer' AND 'united states' AND ('social determinants of health' OR 'medicaid' OR 'medicare' OR 'medically uninsured' OR 'food assistance' OR 'food insecurity' OR 'ill-housed persons' OR 'public housing' OR 'housing instability' OR 'social welfare' OR 'unemployment' OR 'poverty' OR 'socioeconomic factors')
